# Characterizing Urban Household Waste Generation and Metabolism Considering Community Stratification in a Rapid Urbanizing Area of China

**DOI:** 10.1371/journal.pone.0145405

**Published:** 2015-12-21

**Authors:** Lishan Xiao, Tao Lin, Shaohua Chen, Guoqin Zhang, Zhilong Ye, Zhaowu Yu

**Affiliations:** 1 Key Lab of Urban Environment and Health, Institute of Urban Environment, Chinese Academy of Sciences, Xiamen, China; 2 Xiamen Key Lab of Urban Metabolism, Xiamen, China; National Cheng-Kung University, TAIWAN

## Abstract

The relationship between social stratification and municipal solid waste generation remains uncertain under current rapid urbanization. Based on a multi-object spatial sampling technique, we selected 191 households in a rapidly urbanizing area of Xiamen, China. The selected communities were classified into three types: work-unit, transitional, and commercial communities in the context of housing policy reform in China. Field survey data were used to characterize household waste generation patterns considering community stratification. Our results revealed a disparity in waste generation profiles among different households. The three community types differed with respect to family income, living area, religious affiliation, and homeowner occupation. Income, family structure, and lifestyle caused significant differences in waste generation among work-unit, transitional, and commercial communities, respectively. Urban waste generation patterns are expected to evolve due to accelerating urbanization and associated community transition. A multi-scale integrated analysis of societal and ecosystem metabolism approach was applied to waste metabolism linking it to particular socioeconomic conditions that influence material flows and their evolution. Waste metabolism, both pace and density, was highest for family structure driven patterns, followed by lifestyle and income driven. The results will guide community-specific management policies in rapidly urbanizing areas.

## Introduction

Waste generation and resource shortages have long been recognized as two of the greatest challenges facing human society[1]. Urban metabolism is the sum of the technical and socio-economic processes resulting in growth, energy production, and waste elimination [2]. Solid waste is a major product of urban metabolism, accounting for 30% of the total material input[3]. Waste metabolism is part of urban metabolic process and threatens the sustainability of cities, indicating that urbanization is accelerating entropy with no promising resolution in the near future[4–5]. However, waste is a good indicator of urban function during in urban sprawl when comparing multi-scale urban metabolisms to guide the development of public policies[6].

Waste management is one of the most important services provided by a city, and the effectiveness of waste management directly affects the sustainability of a city[7]. As populations are concentrated in cities, waste management becomes an increasingly complex challenge involving psychological, political, and economical factors[8]. With the unprecedented spread of urbanization, China is undergoing a rapid growth rate of municipal solid waste. Currently, China is the world’s largest waste generator, and solid waste management involves numerous environmental and administrative challenges [9–10]. A framework of waste management strategies aiming to reduce waste and promote recycling was promulgated by the Chinese central government in 2008 to alter solid waste generation habits and waste disposal activities in China. However, the strategies had limited success despite considerable financial investment from the government[11]. Municipal solid waste has increased from 154 million tons in 2008 to 171 million ton in 2012. Waste mismanagement has transformed environmental problems into social conflicts in China. Waste management systems are on an unsustainable trajectory in the world’s largest waste-generating country.

So far, studies on waste management have focused on the factors influencing waste generation in both developed and developing countries[12–15]. Information about relevant influential factors is essential to predict the consequences of changes in economic systems, demographics, and policy measures on future waste generation[16]. Income and family size are highly cited as major determinants affecting solid waste generation. Other factors such as population density, education, family structure, lifestyle, geographic features, and policies also inevitably influence waste generation and composition[17–23]. Waste management is a complex eco-social system that is shaped by these factors[24]. Researchers have also recognized that rapid urbanization, soaring inequality, and varying culture and institutional issues have complicated waste management in developing countries. Thus, waste management solutions in developed countries are not sufficient, and are even counterproductive to solving waste issues in developing countries[24–25]. The analysis of influential factors is normally a stationary determinant that cannot effectively explain how geographic and demographic factors influence waste generation and metabolism in a changing society.

In the context of rapid urbanization, China has transformed from one of the most egalitarian countries to one with the highest level of social inequality[26]. According to the World Bank, the Gini index has risen to 0.42 in 2009 in China, higher than the warning level set by the United Nations, while the index was only 0.29 in 1981. It is obvious that Chinese society is stratifying. China’s rapid urbanization process has resulted in diverse urban communities, which represent an appropriate scale to capture the complexity of dynamic systems[27–28]. In terms of waste management, many efforts have been made to explore the relationships between solid waste generation and socioeconomic factors at the household or national level. The complexity of waste management has recently increased, accompanied by growing scrutiny from local communities[29]. However, less attention has been paid to communities, which are a basic unit for waste management in China. Currently, accessible waste data at the community level is scarce, which makes it difficult to address the causes and effective solutions of the problem. It is imperative to advance our understanding of waste generation patterns and metabolism in transitional stratified societies. A better understanding of waste generation in relation to community stratification will enable cities to develop specific sustainable waste management policies. In this paper, we specifically address the following questions using a well-designed household waste survey: (1) Does household waste generation vary among urban communities? (2) If so, how does community stratification influence waste generation and metabolism? We believe that a holistic consideration of varying socioeconomic factors due to rapid urbanization will supply important information for guiding effective and responsive waste management.

## Methods

### Study area and spatial sampling of targeted communities

**Fig 1:**
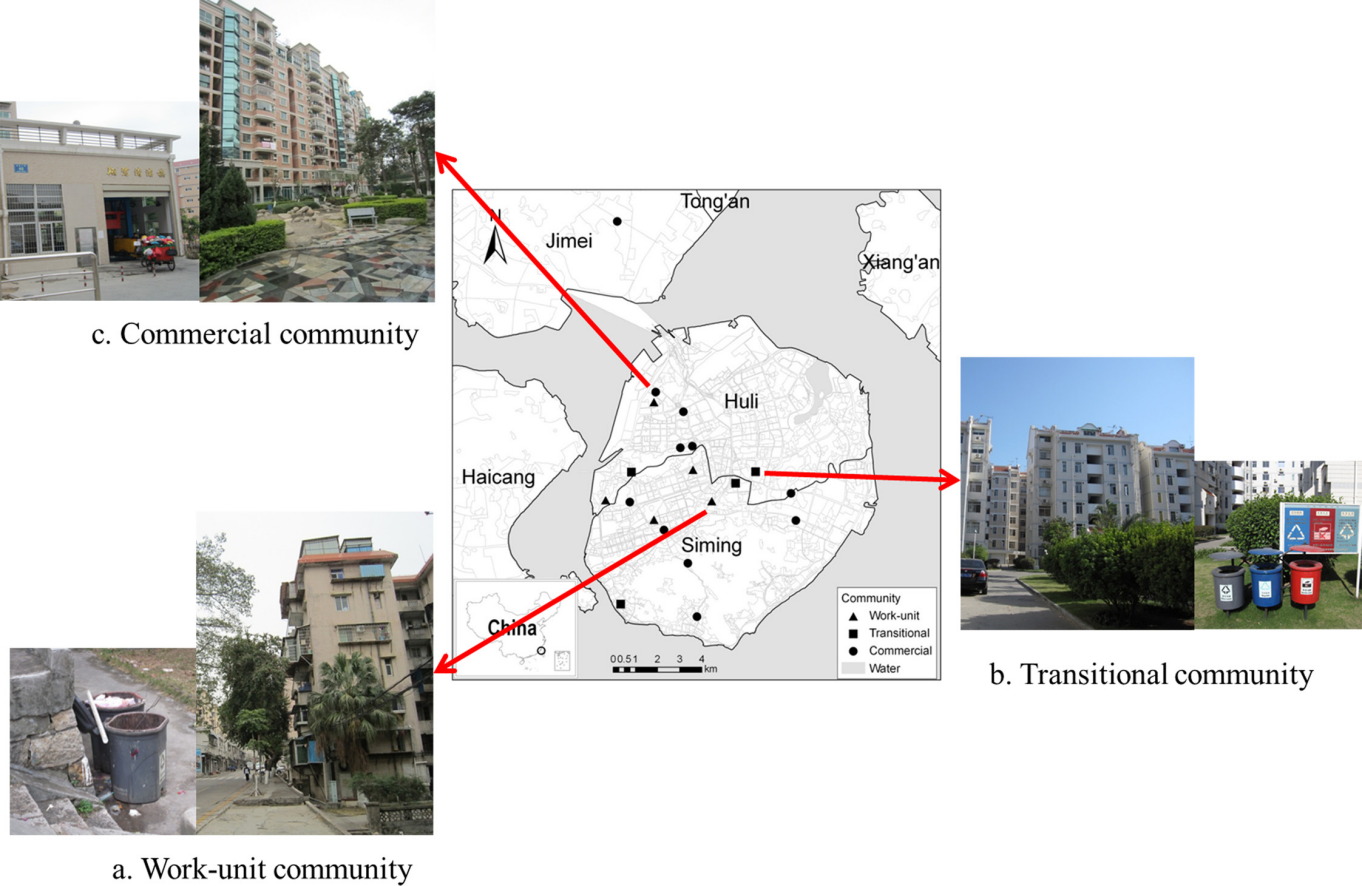
Location of communities in which sampling was conducted.

The research was conducted in Xiamen, a rapidly urbanizing coastal city in southeast China (Fig 1). The terrestrial area is 138.41 km^2^ and the resident population is 1.9 million in Xiamen Island. Since its establishment in the 1980s as one of five Special Economic Zones (SEZs) in China, Xiamen has undergone rapid economic development and urbanization. The gross domestic product per capita reached 11 thousand US dollars in 2012, about 20 times higher than that when the SEZ was initially established in 1980. Over the last two decades, the urbanization rate has steadily increased from 38.5% to 80.5%, and the built-up area gradually has expanded by a factor of 2.5 (Xiamen Statistical Bureau, 2012). Municipal solid waste surged from 240,000 tons in 1996 to 810,000 tons in 2012.

We applied a multi-object spatial sampling method to obtain representative communities. This method combines spatial statistics with GIS techniques to minimize the sampling error and improve sampling efficiency in view of the heterogeneous spatial demographics of households and residential communities[30]. GIS is a robust tool for analyzing various factors and for importing, managing, and analyzing spatial data[31]. In this study, the community characteristic factors included construction conditions (building density, plot ratio), residential land price, population density, and landform[32]. Nineteen communities from the Island and one community from the off-island area were selected to trace the current spatial urban growth of Xiamen City (Fig 1).

Chinese cities have undergone a housing policy reform characterized by a change from a welfare-oriented to a market-oriented housing distribution system. This reform began in 1984, and it has had a significant influence on community type[33–36]. The selected communities were classified into three types in the context of the housing policy reform in accordance with their construction time. Work-unit is a generic term denoting socialist work places in China, which provided employees a comprehensive package of welfare and services including housing before the housing policy reform[37]. Five of the selected communities are work-unit communities. They were built before the reform in the 1980s and are characterized by the Soviet architectural philosophy, low-rise and brick concrete structures, with little open space and poor infrastructure. Another four communities are considered transitional types. They were built between the late 1980s and mid-1990s, when the housing distribution system included a mix of market-trading and government regulation. The other 11 communities are commercial communities built after the mid-1990s. These commercial communities are characterized by high building densities and multistory or high-rise buildings. The living standard in the commercial communities is better and features attractive landscapes and excellent facilities and services. The locations of the selected communities are shown in Fig 1. The pictures indicated by arrows show the building façades and waste collection facilities in the three community types. No waste management service other than curbside collection is provided in work-unit communities (Fig 1A). Waste is always collected in the front of buildings in transitional communities (Fig 1B). In commercial communities, property management provides waste services, with door-to-door service or onsite community collection, as well as a waste transfer station (Fig 1C).

### Demographic information and household waste collection

Approximately 10 households were randomly selected in each of the 20 communities. A total of 191 households granted us permission to conduct the waste generation and physical composition survey, and at the same time, demographic information was acquired through questionnaires (Table 1). All the households voluntarily sorted household waste into three categories (i.e., food, recyclables, and other waste).

**Table 1:** Components and survey variables in the questionnaire.

Component	Survey variable
Homeowner	Income, age, sex, education, religious affiliation, occupation
Other family members	Age, education, religious affiliation, occupation
Entire household	Income, size, living area, frequency of eating at home

A description of our research purpose and use was provided in the first part of the questionnaire. The participants were informed before the survey that the results would only be used in an aggregated form that made it impossible to identify individuals, and therefore protected the privacy of the participants. All participants provided us oral consent to participate in this study. If they did not agree to participate in the survey, they rejected it at the beginning. It was impossible to obtain information from the survey without the participant’s consent. The Academic committee of the Institute of Urban Environment (IUE) is an ethics committee and has approved the consent for it is a regular procedure. We anonymized all personal demographic data prior to analysis. Our study obtained ethical approval from the Academic Committee of IUE.

Research staff collected household waste during a period of seven consecutive days from March 29 to April 4 2012. Datasets of waste generation and physical composition for each household were obtained by manual separation and weighing immediately after collection, according to the Sampling and Analysis Methods for Domestic Waste (CJ/T 313–2009) enacted by the Ministry of Housing and Urban-Rural Development, China. According to the standard methods, the physical composition of domestic waste was determined by manual sorting The waste was sorted into 11 categories, including food waste, paper, rubber and plastic, textiles, gardening waste, dust, ceramic and brick, glass, metal, hazardous waste, and mixed waste. Mixed waste was undersized residue sorted with a 10-mm mesh sampling sieve, and which was difficult to further subdivide. The separation and weighing processes were conducted in public places, which did not contain endangered or protected species. No specific permission was required for these locations/activities.

### Multi-scale integrated analysis of societal and ecosystem metabolism

A multi-scale integrated analysis of societal and ecosystem metabolism (MuSIASEM) was used to analysis the complex system of urban metabolism. Barles [6] emphasized the necessity to link metabolism to particular socioeconomic conditions that influence material flows and their evolution. MuSIASEM defines the intensity of material and energy flows in land and time dimensions[38]. Thus, it is possible to apply this method to sustainability science or urban metabolism by investigating the links between social activities and environments or resources[39]. This method is effective in analyzing metabolism with respect to urban strategies, economic development, environmental policy, and social transformation at national and regional scales[40–43]. Waste metabolism determined by MuSIASEM is a new tool to enrich information on waste patterns[41]. Waste metabolism is calculated using the following formulas with a fund-flow representation.

THA=FS×24

$$HA=THA-HA_{pw}-HA_{edu}-HA_{eo}$$

$$WMR=\frac{WG}{HA}$$

$$DW=\frac{WG}{LA}$$

Where, THA is a fund element of total human activity, representing the total hours of a household in one day; FS is family size; HA is a fund element of human activity, representing the total amount of hours spent at home including household chores and physiological overhead; HApw is the total labor hours in paid work sectors for one day; HAedu is the education hours for children; HAeo is the time spent eating out; LA is a fund element, representing the total living area for a household; WG is a flow element, representing the total waste generated in a household; WMR is the waste metabolic rate, indicating the pace of waste generated per human activity, measured in kg/h; and DW is waste density, indicating the waste generated per day in a given area, measured in (kg/d)/ m^2^.

## Results and Discussion

### Household waste generation and composition

**Fig 2:**
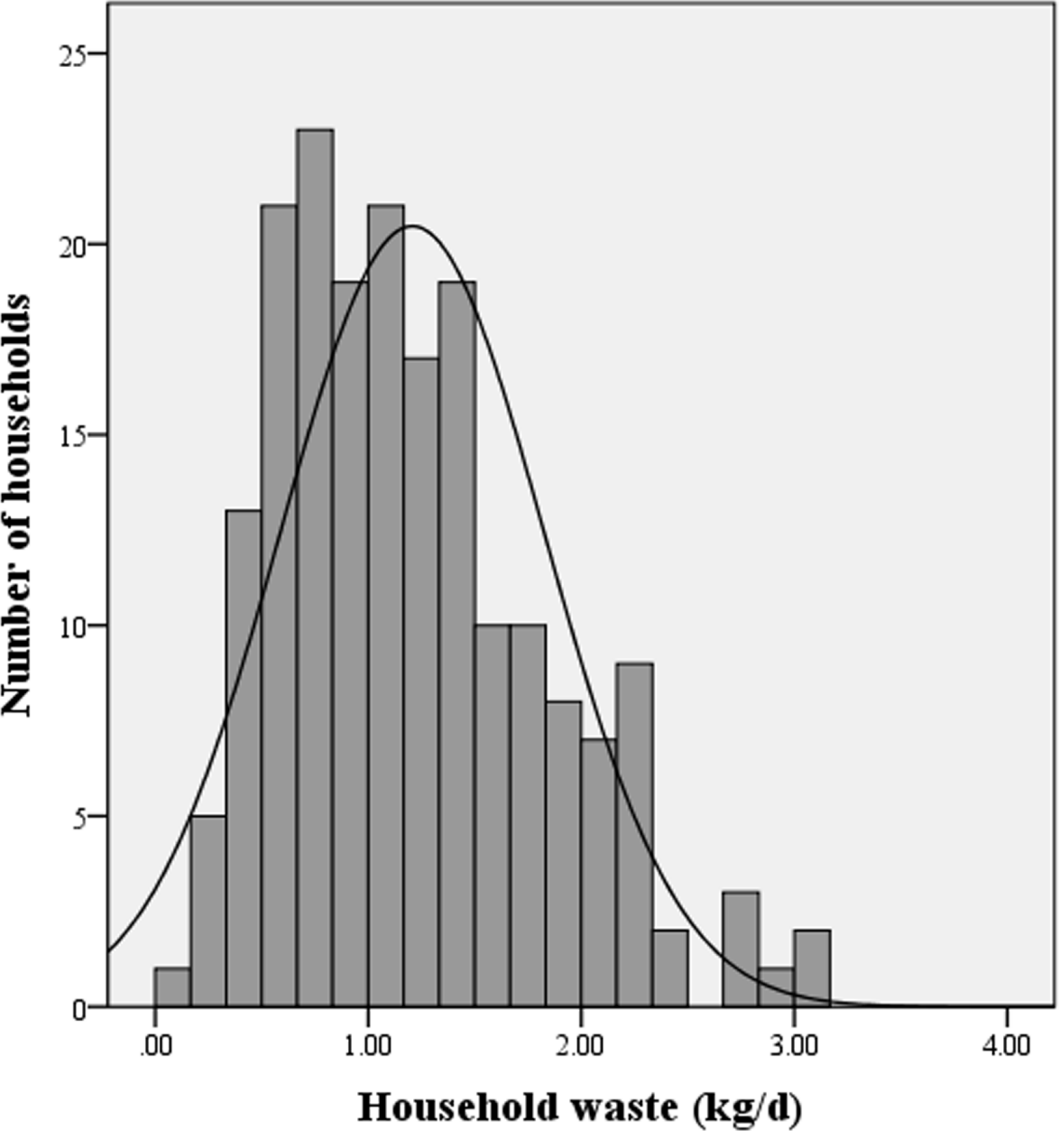
Household waste distribution.

**Fig 3:**
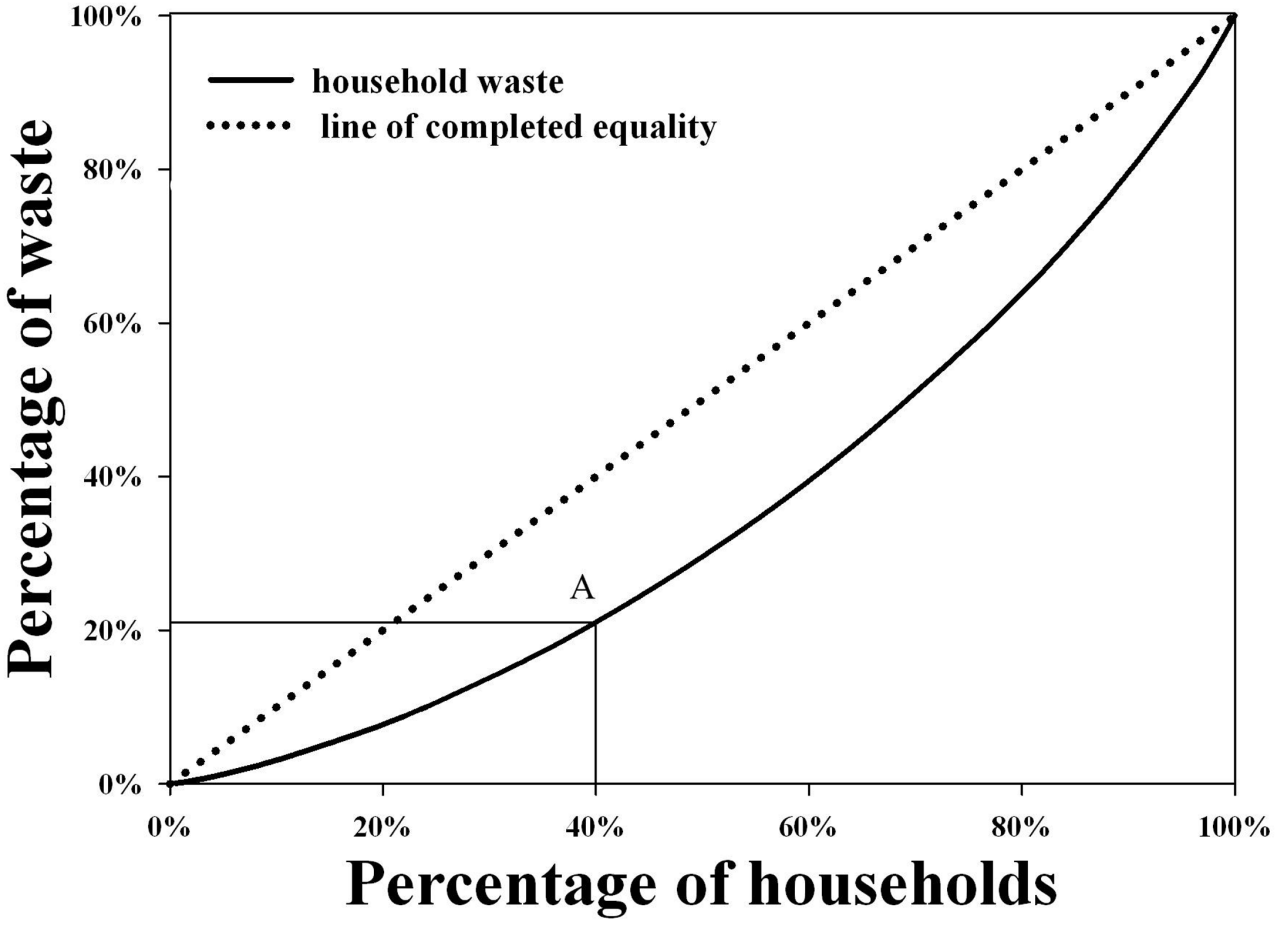
Lorentz curve of household waste generation.

The average household waste generation in Xiamen City was 1.21 kg/d for each household and 0.35 kg/d per capita. During the survey, the weight of waste varied from 0.15 to 3.09 kg/d and from 0.07 to 1.05 kg/d per capita (Fig 2). The distribution of household waste generation was positively skewed (Table 2). Fig 3 shows the Lorenz curve of waste generation. The curve ranks all households from lowest to highest contributors. It describes the cumulative percentage of household contributions to waste generation. Point A demonstrates that about 40% of the sampled households were responsible for approximately 20% of the total waste generation. The dashed line indicates the point at which each household contributes equally to the total waste. The Gini coefficient was calculated based on the Lorenz curve. The Gini coefficient, a ratio between 0 and 1, is a commonly used economic measurement of income inequality or wealth distribution. This method has been applied in environmental science to assess the equality of pollutant discharge such as wastewater and carbon emissions[44–45]. The Gini coefficient measured during the survey was 0.30. There are no conflicts in household waste discharge in the city, and thus residents have the same waste discharge rights. The Gini coefficient illustrates significant inequality among households in the survey. The waste supports the notion that there are divergent factors leading to differences in household waste generation and metabolism.

**Table 2:** Main parameters of household waste generation distribution (kg/d).

Parameter	Mean	SD	Skewness	Skewness SE	Kurtosis
Household waste generation	1.21	0.62	0.74	0.18	0.18

**Fig 4:**
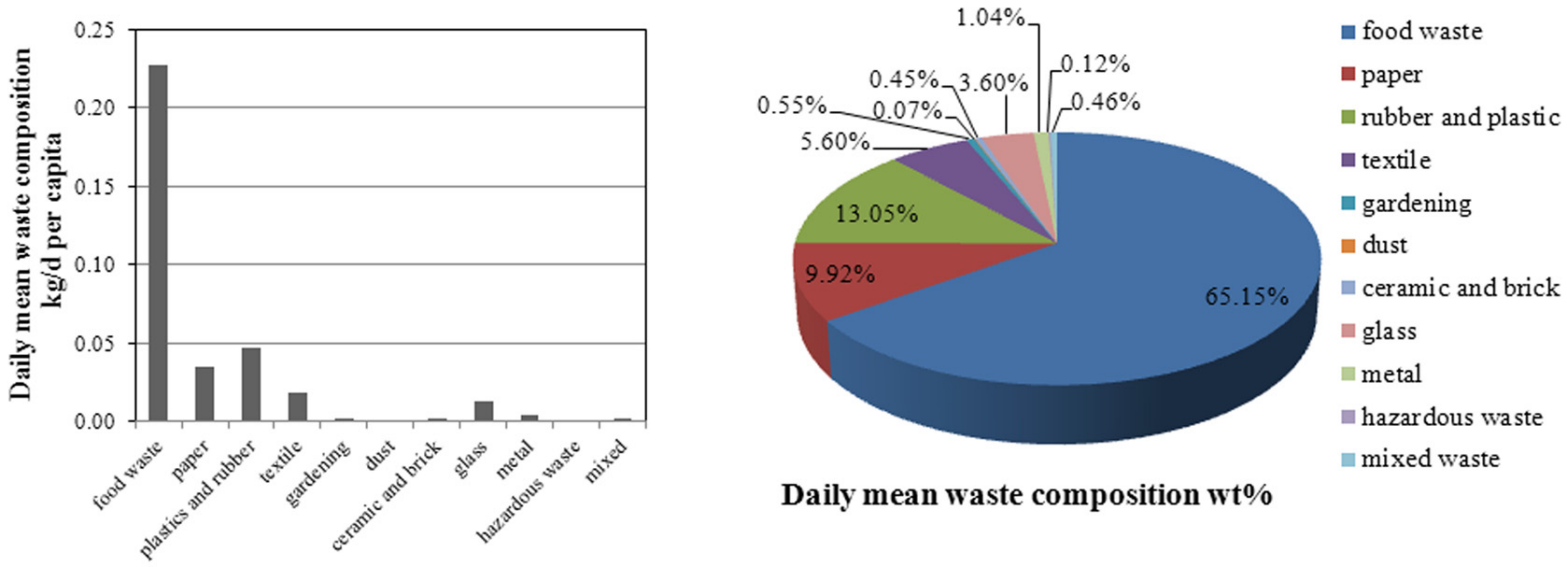
Daily mean waste composition.

Fig 4 depicts the composition of Xiamen household waste. The proportion and weight of each waste type were also determined. Food waste comprised the highest proportion of total waste with 65.1% (0.227 kg/d per capita), followed by plastics and rubber (13.0%,0.047kg/d per capita), and paper (9.9%, 0.035kg/d per capita). Dust, metal, ceramic, and brick had the lowest concentrations, making up less than 1% wt. Our results show that urban household food waste is a major source of Xiamen municipal solid waste. Therefore, the composition of Xiamen household waste highlights the great potential for waste reduction by recycling food waste, plastics and rubber, and paper in the near future.

### Community stratification and household waste generation

#### Household socioeconomic characteristics among different communities

**Fig 5:**
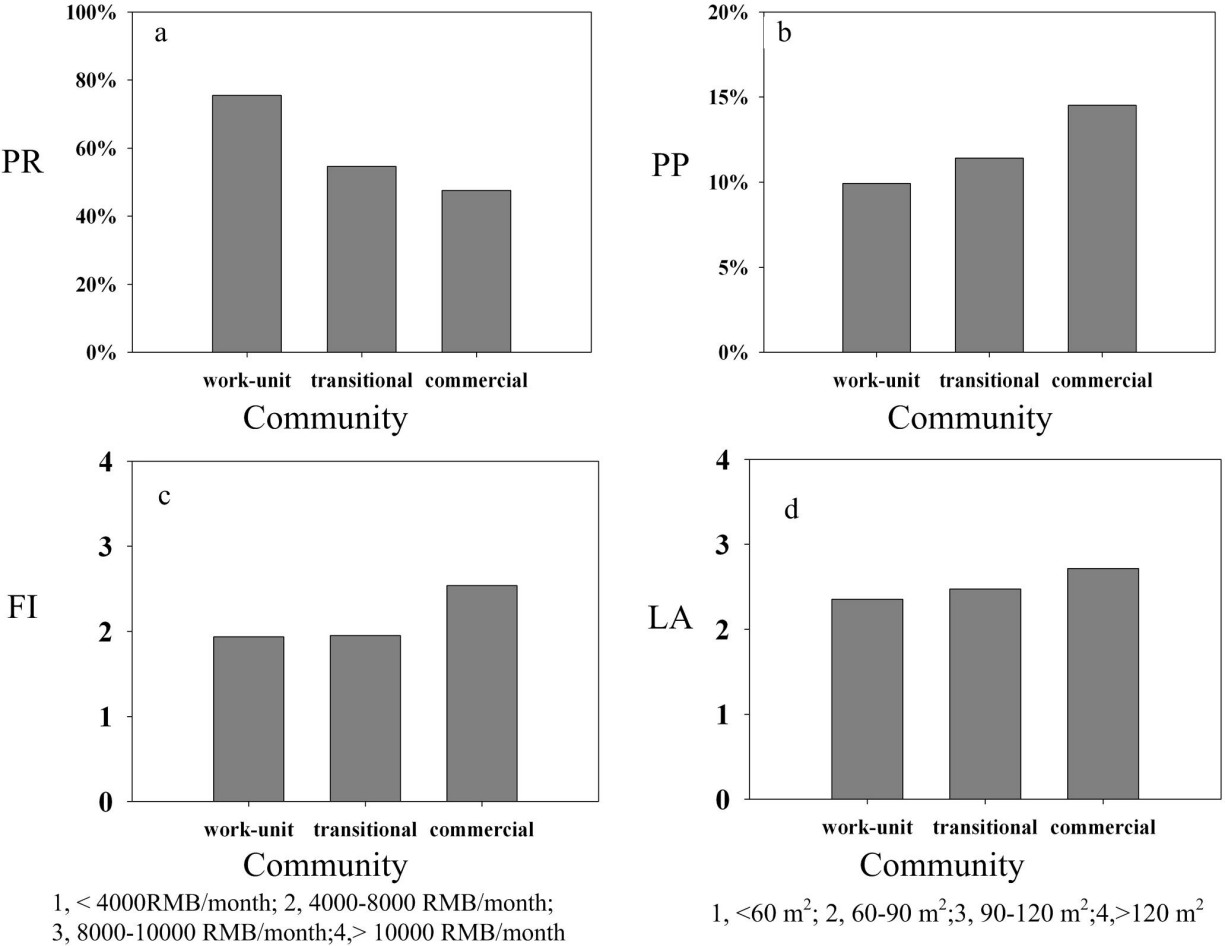
Household characteristics in different community types.

Analysis of variance (ANOVA) was applied to measure differences among the survey variables with the null hypothesis that the distributions of household characteristics were the same across community types. The null hypothesis was rejected when the significance value (*p*)was less than 0.05.A clear differentiation existed among the work-unit, transitional, and commercial communities, generally represented by family income, living area, percentage religious affiliation, and homeowner occupation (Table 3, Fig 5). The percentage of households with a family income below 4000 RMB per month was 44.7%, 42.5%, and 24.6% in the work-unit, transitional, and commercial communities, respectively. It is obvious that commercial community households have an overwhelming economic advantage over households in other communities. Accordingly, they enjoy the largest living area, followed by transitional communities, and work-unit communities. Households with relatively lower family income live in work-unit communities, which were built three decades ago. In contrast, high-income households can afford to purchase modern commercial houses with higher quality surroundings and services. Commercial communities attract more residents working in the private sector, who can afford commercial houses at market rates. In work-unit communities, 75.6% indicated a religious affiliation, but this number decreased to 54.5% in transitional communities, and 47.5% in commercial communities.

**Table 3:** ANOVA of household characteristics in three community types.

									Family structure				
Household Characteristics	FZ	HT	LA	FI	PR	HE	FE	PP	NS	NM	NY	NJ	NC
*p*	0.331	0.167	**0.046**	**0.000**	**0.002**	0.050	0.185	**0.048**	0.704	0.916	0.551	0.494	0.290

FZ, family size; HT, housing tenure; LA, living area; FI, family income; PR, percentage of people indicating religious affiliation; HE, percentage of people with higher education; FE, frequency of eating at home; PP, percentage of people working in private sector; NS, number of senior people (>61 year old); NM, number of middle-aged people (36–60 year old); NY, number of young people (19–35 year old); NJ, number of juveniles (7–18 year old); NC, number of children (0–6 year old).

*p*< 0.05 indicates household characteristics significantly differ among the three community types.

#### Household waste generation among the different communities

**Fig 6:**
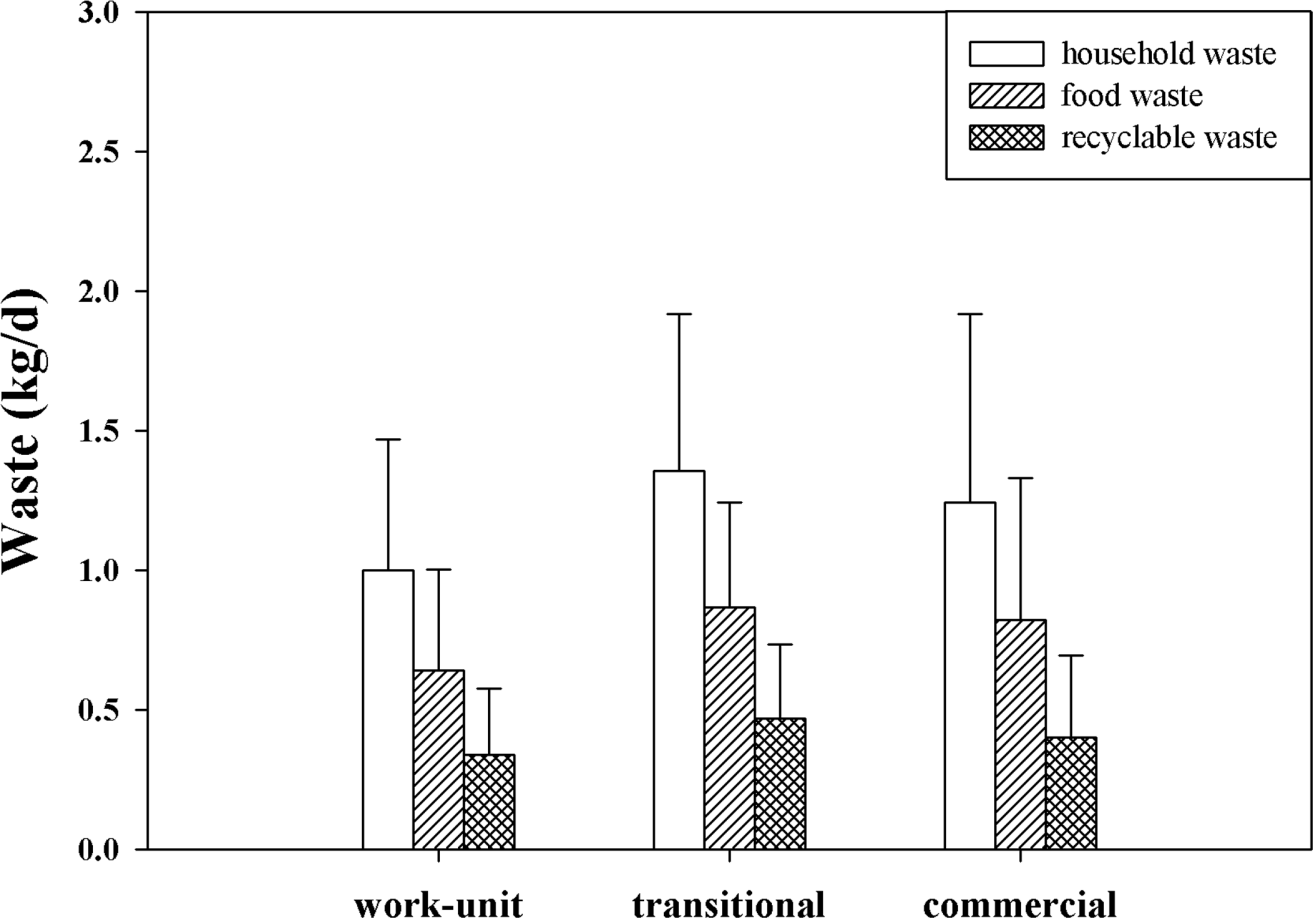
Waste generation in different community types.

Households in transitional communities produced the largest amount of household waste, followed by commercial communities, whereas work-unit communities generated the lowest amount of waste. A similar trend was also observed for food waste and recyclable waste generation. Food and recyclable waste accounted for more than 95% of the household generation. Table 4 shows the significance value of household waste and its composition among the three community types determined by ANOVA. Household waste and its majority composition (food waste and recyclable waste) significantly differed among the three community types (Fig 6). The influence of community stratification on waste generation is further explored in the next section.

**Table 4:** ANOVA results of waste generation and composition among three community types.

Waste type	Household waste	Food waste	Recyclable waste	Paper	Rubber and plastic	texile	Gardening waste	Dust	Ceramic and brick	Glass	Metal	Hazardous waste	Mixed waste
*p*	**0.018**	**0.029**	**0.020**	0.052	**0.011**	0.354	0.126	0.228	0.258	0.245	0.641	0.464	0.750

*p*< 0.05 indicates waste generation and composition significantly differ among the three community types.

### Drivers of household waste generation considering community stratification

#### Influential factors of household waste generation

The results of a correlation analysis show that family size, living area, frequency of eating at home, and family structure were significantly related with household waste generation, whereas household tenure, education, religious affiliation, and homeowner occupation had no significant relationship with waste generation. The strongest relationship existed between waste generation and frequency of eating at home (r = 0.405, *p* < 0.05) and family size (r = 0.276, *p* < 0.05).

It is reasonable that a higher frequency of eating at home and bigger family size correlate to more household waste. However, an inverse relationship between waste production per capita and family size was apparent. Single-person households generated, on average, about three times as much waste per capita as those with six or more persons, about twice that of 4-person households, and about 75% more than 3-person households. As family size increases, the frequency of eating at home increases. Four-person households eat at home 70.65% more often than singe-person households. Smaller family households consume more processed and prepared food, indicated by packaging waste generation. Consequently, paper consumption increases as family size increases. Paper consumption significantly differs among families of different size (ANOVA; *p* = 0.012). Previous studies have emphasized the influence of income on waste generation[41]. Yet, our case is different in that a statistically insignificant relationship was found. Our results show that medium income (4000–15000 RMB/month) households produced the largest amount of household waste compared with low (< 4000RMB/month) and high income (> 15000RMB/month) households. These findings are consistent with the Environmental Kuznets Curve (EKC) theory, which is the hypothesized relationship that environmental degradation tends to worsen with economic growth until an average income is reached over the course of development. The theoretical framework and empirical study of the waste Kuznets curve (WKC) incorporate waste generation and economy, though the evidence for WKC is still contested. Nevertheless, EKC is a reduced model that does not specify the links between economy and environmental pollution. Extended studies have explained this relationship as a mixed result of scale, composition, and technology effects or IPAT identity[46]. There is still a need to explore the reason for the shape of the WKC for specific situations. Therefore, in the next section, we use a regression model to understand the underlying causes of household waste generation. Although 56% of respondents indicated a religious affiliation of Buddhism, waste generation differences between those with and without religious affiliation were marginal. Personal values and charitable motivations are assumed to be drivers for material reuse in waste prevention studies [47]. However, respondents with religious affiliations in our survey did not produce lower waste as expected. This result is similar to that found in Bangladesh, in which 90.7% of respondents were Muslim [48].

#### Differentiated household waste generation patterns

All of the factors influencing household waste generation in Table 5 are related to social differentiation, which always results in social inequality especially in environmental issues [49–51]. A stepwise regression was applied to eliminate multi-collinearity and identify the drivers of household waste generation considering community stratification (Table 6). The results confirm that the determinants of household waste generation varied among the three community types. Only the frequency of eating at home and family structure corresponded to household waste generation without considering community stratification. However, different and detailed waste generation patterns emerge when considering community stratification. In work-unit communities, though both frequency of eating at home and family income had positive effects on household waste generation, the latter had a stronger effect. Decoupled signals of income and waste generation were detected in transitional and commercial communities. In transitional communities, homeowner occupation and number of children were the main factors influencing household waste generation. In the regression model, the number of children had a robust and positive coefficient, making the variable more decisive. In terms of commercial communities, the frequency of eating at home was a prominent factor and the only statistically significant one influencing waste generation. The results indicate that the consideration of community stratification is necessary for acquiring detailed information on household waste generation patterns.

**Table 5:** Correlations between waste generation and family characteristics.

									Family age structure				
Factors	FZ	HE	LA	FI	PR	FE	HE	HO	NS	NM	NY	NJ	NC
r	**0.276**	-0.028	**0.179**	0.098	0.038	**0.405**	0.032	0.072	0.104	0.018	0.108	0.021	**0.220**
*p*	**0.000**	0.697	**0.013**	0.182	0.598	**0.000**	0.662	0.325	0.153	0.802	0.137	0.774	**0.002**

**Table 6:** Multiple regression models of drivers of household waste using stepwise regression.

Community	Model	R^2^	DW-value
Total household scale	Y = -0.585***+0.201NC*+0.010FE***	0.46	1.71
Work-unit community	Y = -0.984***+0.009FE**+0.171FI*	0.56	2.21
Transitional community	Y = -0.569**+0.093PP***+0.425NC**	0.56	2.17
Commercial community	Y = -0.663***+0.012FE***	0.49	1.83

Y is the natural logarithm of household waste.

*p < 0.05

** *p* < 0.005

*** *p* < 0.0005.

The DW-value is the result of the Durbin–Watson statistic test used to detect the presence of autocorrelations (relationship between values separated from each other by a given time lag) in the residuals in the multiple regression model. In general, the DW value is approximately equal to 2 when there is no statistical evidence that the error terms are autocorrelated.

The stepwise regression model facilitates the understanding of how dynamic factors, which incorporate socioeconomic conditions, affect waste generation in a rapidly urbanizing city. Households in work-unit communities are characterized by low family income and poor living conditions. The results of the work-unit communities are in line with previous studies that have found that the waste generation rate increases as family income increases. The model reflects a positive elasticity of the waste generation to income ratio (0.171, in the range 0.11–0.23) according to Mazzanti and Zoboli (2009)[20]. The waste generation pattern in work-unit communities can therefore be summarized as income driven. In transitional communities, nearly one-third of the homeowners are retired. Three-generation extended families require more materials to satisfy their needs, and thus produce the most waste. To eliminate the influence of family size, the natural logarithm per capita waste generation was considered as a dependent variable. The independent variables remained the same. This regression model shows that NC is still significant in the regression model. The result further confirms that family structure, rather than family size, is a key underlying factor in determining household waste in transitional communities. The waste generation pattern in transitional communities can be summarized as family structure driven. In commercial communities, the frequency of eating at home is the only factor positively related to household waste generation. Different consumption activities produce different amounts of waste [14]. Households in communities with the highest family income are inclined to leisure consumption and produce the least waste at home. A significant and negative correlation was found between family income and the average frequency of eating at home (r = -0.230, *p* < 0.005), indicating that people tend to eat out more when they have higher purchasing power. As food waste comprises the largest proportion of household waste, eating habits have a major influence on waste generation. The household waste generation pattern in commercial communities can be summarized as lifestyle driven.

Community transition will continue to accelerate as urban renewal and ‘old-town’ reconstruction persists in the future in China. The proportions of work-unit and transitional communities will gradually decrease and transform into commercial communities. The urban household waste generation pattern in Chinese cities is very likely to be altered from income driven to family structure driven, and then to lifestyle driven.

### Household waste metabolism by MuSIASEM

**Fig 7:**
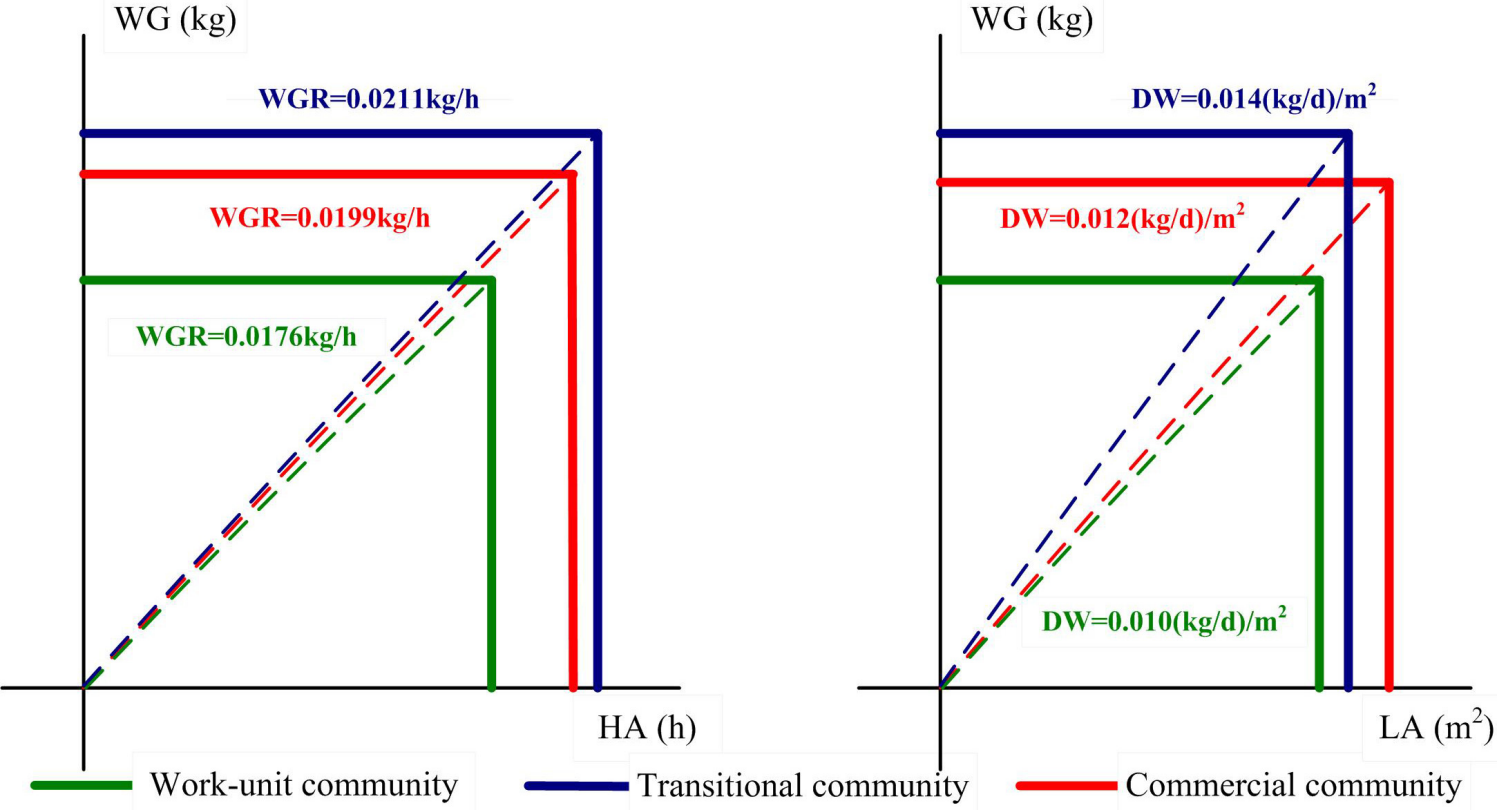
Flow-fund representation of household waste.

To visualize waste metabolism, we used a single definition of flow waste generation and two definitions of fund HA (human activity) and LA (living area) to make a flow-fund representation (Fig 7). The two fund variables provide a vivid description of the socioeconomic characteristics of the three communities. In work-unit communities with low family income, people spent less time (56 hours) at home and more time working. The term WMR was obtained by dividing waste flow by the hours of human activity. WMR was much lower in work-unit communities compared with that in the other communities. People in transitional communities spent the most time (65 hours) at home owing to family structure (i.e., more seniors and children). However, long human activity time at home cannot compensate for the high level of waste generation, and the WMR (0.0211kg/h) was still the highest in transitional communities. The WMR was relatively lower in transitional communities because people spent more time eating out. The ranking of DW was the same as for WMR. The DW in work-unit communities was the lowest in terms of poor living conditions. The DW in commercial communities ranked second due to the tradeoff between living area and waste generation. The family structure driven pattern led to the largest WMR and DW, whereas the income driven pattern led to the lowest WMR and DW. The MuSIASEM approach provides an analytical method to account for time use and living conditions for different family types and integrates these factors with community waste generation.

Many previous studies have highlighted that waste reduction requires extensive community participation[11],[52]. However, limited research has addressed the differences in waste generation patterns and metabolism with respect to community stratification and its policy implications. Waste metabolism is controlled by complex interactions between society and the environment. To adopt different measures targeting households in different community types, urban household waste reduction policies in Chinese cities require a full understanding of the current and future development of socioeconomic stratification. Cities typically adopt a one-size-fits-all approach to waste management. In reality, local community is a major factor for translating city-wide management to local practices. With the highest waste metabolism rate and density, households in transitional communities should be the preferential group for a stringent waste sorting and reduction policy. Meanwhile, environmental education programs should target households in commercial communities to promote environmentally friendly lifestyles. For households in work-unit communities, improvements to waste infrastructure and services are urgent for achieving waste reduction. Linking our results within the context of waste management, we argue that implementing community-specific management practices rather than the current one-size-fits-all framework is key for the success of waste management in Chinese cities.

## Conclusions

In this study, we investigated household waste generation and composition in 20 urban communities in Xiamen. The paper explores waste generation driving patterns and waste metabolism considering community stratification. Multiple regression models and MuSIASEM were successfully applied to study waste generation patterns and metabolism at the community scale. These methods allow us to better understand the complexity of urban sustainability and identify causal relations, which are often hidden.

The results show that there are considerable differences in waste generation among urban households. The distribution of waste generation is skewed, indicating that a small number of urban households contribute to a disproportionately large fraction of total household waste. Household waste generation is closely related to a few social economic conditions. Rapid urbanization in China has resulted in community stratification. Waste generation patterns differ among work-unit, transitional, and commercial communities, leading to diverse waste metabolism. The pace and density of waste generation are highest in family structure driven communities, followed by lifestyle and income driven.

Community transition will continue to accelerate in China as urban renewal and ‘old-town’ reconstruction persist in the future. The proportions of work-unit and transitional communities will gradually decrease and transform into commercial communities. Household waste generation and metabolism will change in line with community transition, and the waste generation pattern is expected to evolve from economic driven to family structure driven, and eventually to lifestyle driven. The consideration of community stratification is important for effectively exploring household waste generation patterns in urbanizing cities. These results bring to light the different responses of communities. This paper highlights the waste generation based survey approach, which presents accessible data to address the causes of waste generation and its disparity. Our results will help to identify efficient and equitable targeted measures for waste reduction by differentiating policies among community types.
